# Thyroid Storm and Type 1 Diabetes Mellitus Induced by Combined Ipilimumab and Nivolumab Immunotherapy: A Case Report

**DOI:** 10.7759/cureus.46985

**Published:** 2023-10-13

**Authors:** Vivek Ramburuth, Jeyanthy Rajkanna

**Affiliations:** 1 Internal Medicine, Peterborough City Hospital, Peterborough, GBR; 2 Endocrinology and Diabetes, Peterborough City Hospital, Peterborough, GBR

**Keywords:** immune checkpoint inhibitors, thyroiditis, nivolumab, ipilimumab, type 1 diabetes mellitus (t1dm), thyroid storm, combination therapy

## Abstract

Immune checkpoint inhibitors have revolutionised the management of cancer, and they are being used in combination to improve survival outcomes. Combination therapy is, however, associated with an increase in the frequency and severity of immune-related adverse events such as endocrine disorders. We report a case of simultaneous onset thyroid storm and type 1 diabetes mellitus induced by ipilimumab and nivolumab therapy in a patient with advanced melanoma. This case report suggests that combination immunotherapy can trigger a robust immune reaction leading to the development of multiple life-threatening endocrinopathies, including rapid onset destructive thyroiditis and insulitis. Prompt identification and management are essential to prevent morbidity and mortality.

## Introduction

Immune checkpoint inhibitors (ICI) have emerged as a promising therapeutic approach for the management of various cancers that were previously associated with a poor prognosis. They are monoclonal antibodies that block the function of immune checkpoints, thereby enhancing T-cell-mediated anti-tumour response [1]. Examples include ipilimumab, which blocks cytotoxic T-lymphocyte 4 (CTLA-4) and nivolumab, which inhibits human programmed death receptor-1 (PD-1). These agents are increasingly being used synergistically to enhance the anti-cancer response, and combination therapy with ipilimumab and nivolumab has significantly improved the survival of advanced melanoma as well as other cancers [2,3]. These agents can, however, disrupt immune tolerance pathways and are associated with immune-related adverse events involving all organ systems, including the endocrine tissues [4]. Combining these agents significantly increases the frequency and severity of these adverse events compared to monotherapy [5].

We describe a case of simultaneous onset thyroid storm and type 1 diabetes mellitus (T1DM) in a patient with advanced melanoma triggered by combined ipilimumab and nivolumab immunotherapy.

## Case presentation

Medical history and demographics

A 48-year-old man presented to the emergency department with pyrexia, fatigue and palpitations. He was recently diagnosed with metastatic melanoma involving the spleen, left adrenal gland and parietal bone, for which he had received two cycles of ipilimumab and nivolumab combination immunotherapy four weeks prior to the onset of his symptoms. Other past medical history included essential hypertension, and his drug history included amlodipine. He was a non-smoker and drank little alcohol. On examination, he was febrile with a temperature of 38.5°C and tachycardic with a heart rate of 140 beats per minute. Blood pressure was 120/60 mmHg, respiratory rate was 20 per minute and oxygen saturation was 100% on room air. The Glasgow Coma Scale score was 15. He appeared agitated and was sweating profusely. There was no jugular venous distension or pedal oedema. Lung auscultation and heart sounds were normal. There was no thyromegaly, thyroid tenderness or exophthalmos. The electrocardiogram showed atrial fibrillation.

Investigations

His laboratory data are shown in Table 1. Blood investigations indicated normal full blood count, renal function and liver function tests. C-reactive protein was slightly elevated. Thyroid function tests showed a suppressed thyroid-stimulating hormone (TSH) level and significantly elevated free triiodothyronine (free T3) and free thyroxine (free T4) levels, indicating severe thyrotoxicosis. Further tests indicated that he was negative for thyroid-stimulating hormone receptor antibody and thyroid peroxidase antibody. The Burch-Wartofsky Point Scale (BWPS) score was 55, highly suggestive of a thyroid storm.

**Table 1:** Summary of laboratory results TSH receptor antibody: thyroid-stimulating hormone receptor antibody

	Value	Reference Range
Full blood count		
Haemoglobin (g/L)	134	130 - 180
White cell count (10^9^/L)	6.4	4.0 - 11.0
Platelet count (10^9^/L)	233	150 - 400
Blood Biochemistry		
Sodium (mmol/L)	131	133 - 146
Potassium (mmol/L)	4.9	3.5 - 5.3
Creatinine (μmol/L)	51	59 - 104
C-reactive protein (mg/dl)	77	<5
Bicarbonate (mmol/L)	25.0	22 - 29
Thyroid hormones		
Free triiodothyronine (pmol/L)	26.1	3.1 - 6.8
Free thyroxine (pmol/L)	>100	12.0 - 22.0
Thyroid-stimulating hormone (mU/L)	0.06	0.30 - 4.20
TSH receptor antibody (IU/L)	1.6	<2.9
Thyroid peroxidase antibody (IU/ml)	14	<34
Diabetes		
Glucose (mmol/L)	23.0	4.0- 5.4
Haemoglobin A1c (mmol/mol)	47	<41
C-peptide (nmol/L)	0.22	0.37 - 1.47
Glutamic acid decarboxylase antibody (IU/mL)	148.8	<10.0
Islet cell antibodies	Negative	
Endocrine		
Cortisol (nmol/L)	363	270-700
Follicle-stimulating hormone (U/L)	8	2 – 13
Luteinising hormone (U/L)	13	2 - 9
Prolactin (mU/L)	161	<330
Insulin-like growth factor 1 (ng/mL)	93	80 - 209

He was also noted to have elevated blood glucose levels (23 mmol/L) with raised ketones (2.3 mmol/L) but without acidaemia (pH level of 7.35, bicarbonate level of 25.0 mmol/L). His haemoglobin A1c was mildly raised. Additional tests showed low C-peptide levels with positive glutamic acid decarboxylase antibody titres in line with autoimmune T1DM (Table 1). Further hormone testing, including cortisol, follicle-stimulating hormone (FSH) and luteinising hormone (LH) were normal (Table 1). A septic screen, including urine and blood cultures and chest radiography, was unremarkable.

Treatment

The thyroid storm was treated with carbimazole 10 mg daily and the atrial fibrillation was managed with bisoprolol 5 mg daily and edoxaban 60 mg daily. Intravenous insulin infusion and intravenous fluid therapy were given to manage his hyperglycaemia and ketosis. He was then transitioned to subcutaneous insulin with long and rapid-acting insulin analogues. His insulin titration was challenging as he had ongoing hyperglycaemia for several days. His daily insulin dose was 125 units, which corresponded to 0.87 units/kg of body weight. On day seven of admission, his tachycardia and blood glucose levels stabilised, and he was discharged.

Outcome and follow-up

He was followed up in our endocrinology outpatient clinic six weeks later. His symptoms abated but his thyroid function tests were still suboptimal (TSH level of 0.02 mU/L, free T3 level of 5.3 pmol/L, free T4 level of 40.9 pmol/L). His blood sugar levels were, however, well-controlled. He remained on carbimazole, bisoprolol, edoxaban and maintenance insulin injections. Given his good performance status and prognostic benefit, his oncologist decided to resume the third cycle of ipilimumab and nivolumab therapy.

## Discussion

We present a case of multiple endocrinopathies triggered by combined ipilimumab and nivolumab therapy. Endocrine dysfunction affects up to 10% of patients receiving ICIs [6]. The most frequently observed endocrinopathy is hypothyroidism (13.1%), followed by hyperthyroidism (11.0%), hypopituitarism (9.5%), and adrenal insufficiency (4.8%) [7]. ICI-mediated diabetes is less common, affecting 0.2% of patients [8]. This patient developed thyroid disease and T1DM concurrently in a short period after the initiation of combined ICI therapy. On searching several databases, we could not identify similar case reports of simultaneous onset thyroid storm and T1DM mediated by combined immunotherapy. Yonezaki et al. reported a case of ipilimumab and nivolumab-induced thyroid storm with significantly elevated blood glucose levels, which was felt to be related to a worsening of pre-existing type 2 diabetes mellitus rather than autoimmune T1DM, given negative autoantibodies and recovery of insulin secretion after three months [9].

The mechanism of ICI-related endocrinopathies is not well understood. CTLA-4 is a negative regulator of T-cell activation, and PD-1 acts as a physiologic brake on unrestrained cytotoxic T-effector function [10]. Blockage of these proteins by ICI therapy may disrupt immune tolerance pathways, leading to abnormal activation of T-cells and destruction of endocrine tissues [10,11]. Our case report suggests that the synergistic effect of combined PD-1 and CTLA-4 blockade can trigger a robust immune response leading to an abrupt onset of multisystem endocrine disorders. This patient developed rapid-onset severe thyroiditis presenting as a thyroid storm with a BWPS score of 55. He concurrently developed destructive insulitis with a rapid decline in pancreatic beta cell function, leading to severe hyperglycaemia and impending ketoacidosis.

Immune-mediated endocrinopathies can pose a diagnostic challenge. In this case, these endocrine disorders presented with non-specific features and could have been misdiagnosed as sepsis given his oncological history. Interpretation of autoantibodies can also be difficult. Lyer et al. noted that 53% of patients with thyroiditis mediated by combined ipilimumab and nivolumab therapy were positive for thyroid peroxidase antibodies [12]. Thirty to 50% of cases of ICI-induced diabetes mellitus are positive for at least one diabetes-related autoantibody [13,14]. Checkpoint inhibitors can lead to severe adverse events. A systematic review of patients with combined ipilimumab and nivolumab therapy indicated that 30% of the patients had to discontinue treatment due to adverse events and treatment-related death occurred in 0.7% of the cases [7]. Their prognostic benefit may, however, still justify their use. In our patient, despite the high morbidity and mortality associated with thyroid dysfunction and T1DM, combination immunotherapy was continued.

Clinicians should keep a high index of suspicion in diagnosing endocrine disorders in patients receiving combined ICI therapy. Other risk factors include a history of pre-existing autoimmune disease, the presence of autoantibodies and certain human leukocyte antigen (HLA) genotypes [15]. Although the relative risk of developing immune-related endocrinopathies varies with the treatment regimen and patient factors; a screening programme is recommended for all patients receiving ICIs. Baseline levels of TSH, free T3, free T4, thyroid peroxidase antibody, adrenocorticotropic hormone (ACTH), 9 am cortisol, FSH, LH, testosterone or oestrogen, fasting glucose, electrolytes, parathyroid hormone and calcium levels should be checked [16]. These should be repeated every six weeks while receiving ICI therapy [16]. This will allow early recognition and management of ICI-mediated endocrinopathies.

## Conclusions

Oncology patients on combined CTLA-4 and PD-1 checkpoint blockage therapy can develop polyglandular endocrinopathies. These disorders can develop in a short period after the initiation of therapy and present as medical emergencies. Combination ICI therapy significantly increases the risk of developing these adverse events; however, their prognostic benefit may still justify their use. These patients require regular screening for endocrinopathies to allow prompt identification and management.
